# Specific enrichment of microbes and increased ruminal propionate production: the potential mechanism underlying the high energy efficiency of Holstein heifers fed steam-flaked corn

**DOI:** 10.1186/s13568-019-0937-8

**Published:** 2019-12-28

**Authors:** Hao Ren, Xiaodong Su, Hanxun Bai, Yuntian Yang, Hongrong Wang, Zeng Dan, Jinbin Lu, Shengru Wu, Chuanjiang Cai, Yangchun Cao, Xinjian Lei, Junhu Yao

**Affiliations:** 1 College of Animal Science and Technology, Northwest A&F University, Yangling, 712100 Shaanxi People’s Republic of China; 2 College of Animal Science and Technology, Yangzhou University, Yangzhou, 225009 People’s Republic of China

**Keywords:** Steam-flaked corn, Finely ground corn, Rumen microbes, Rumen metabolites

## Abstract

Corn grain has a high starch content and is used as main energy source in ruminant diets. Compared with finely ground corn (FGC), steam-flaked corn (SFC) could improve the milk yield of lactating dairy cows and the growth performance of feedlot cattle, but the detailed mechanisms underlying those finding are unknown. The rumen microbiome breaks down feedstuffs into energy substrates for the host animals, and contributes to feed efficiency. Therefore, the current study was conducted to investigate the ruminal bacterial community changes of heifers fed differently processed corn (SFC or FGC) using 16S rRNA sequencing technologies, and to uncover the detailed mechanisms underlying the high performance of ruminants fed the SFC diet. The results revealed that different processing methods changed the rumen characteristics and impacted the composition of the rumen bacteria. The SFC diet resulted in an increased average daily gain in heifers, an increased rumen propionate concentration and a decreased rumen ammonia nitrogen concentration. The relative abundance of the phylum *Firmicutes* and *Proteobacteria* were tended to increase or significantly increased in the heifers fed SFC diet compared with FGC diet. In addition, the relative abundance of amylolytic bacteria of the genera *Succinivibrio*, *Roseburia* and *Blautia* were elevated, and the cellulolytic bacteria (*Ruminococcaceae_UCG*-*014* and *Ruminococcaceae_UCG*-*013*) were decreased by the steam flaking method. Spearman correlation analysis between the ruminal bacteria and the microbial metabolites showed that the rumen propionate concentration was positively correlated with genera *Succinivibrio* and *Blautia* abundance, but negatively correlated with genera *Ruminococcaceae_UCG*-*014* abundance. Evident patterns of efficient improvement in rumen propionate and changes in rumen microbes to further improve feed conversion were identified. This observation uncovers the potential mechanisms underlying the increased efficiency of the SFC processing method for enhancing ruminant performance.

## Introduction

Cereal grains are the prevailing feed energy source for ruminants in the livestock industry, and have significant economic importance for human consumption (Marshall et al. 2013). Approximately 75% of the corn grain energy value is derived from starch (NRC 2001). The improvement of starch utilization may improve animal health conditions and performance, and reduced feed costs when grain prices are high. Also, high grains utilization efficiency could alleviate the competition between livestock and humans for food availability (Ertl et al. 2016).

Grain processing is the most widely used method to maximize starch digestibility and improve animal performance in the feedlot industry (Zinn et al. 2002). The germ and endosperm are surrounded by the pericarp, and the protein matrix in the endosperm blocks starch granules in corn grain, which collectively impedes the attachment of microbes to degrade starch (Kotarski et al. 1992; McAllister et al. 1994). Steam flaking could gelatinize starch granules, disrupt crystalline structures, and dissolve polysaccharides, thereby enabling the diffusion of compounds from ruptured granules (Safaei and Yang 2017) and allowing ruminal microorganisms increase access to amylose and amylopectin molecules (Kotarski et al. 1992). However, grinding corn, as a conventional and low-cost processing method, is still used for ruminants. Ferraretto et al. (2013) summarized that the total starch digestibility of steam-flaked corn (SFC) was significantly higher than of finely ground corn (FGC), 93.9% vs 92%. Studies have shown that feeding ruminants SFC increases milk performance in cows (Chen et al. 1994; Zhong et al. 2008; Cooke et al. 2008; May et al. 2009; Luo et al. 2017; Miyaji and Nonaka 2018) and the growth performance in feedlot cattle (Lee et al. 1982; Owens et al. 1997; Zinn et al. 2002; May et al. 2010). However, the detailed mechanisms underlying the effect of corn processing methods on animal performance are not fully understood.

The rumen is a large fermentation tank for ruminants and contains diverse bacteria, archaea, protozoa and fungi. The microbial communities are crucial for the degradation of complex feeds into volatile fatty acids (VFAs) and the synthesis of vitamins and protein for ruminants’ health and production performance (Krause et al. 2003; Zilber-Rosenberg and Rosenberg 2008). Many factors affect the ruminal microbial community, such as host (Malmuthuge and Guan 2017), heredity (Paz et al. 2016), diet (Hua et al. 2017), disease (Ma et al. 2018), physical stage (Zhu et al. 2017), age (Jami et al. 2013) or additives (Uyeno et al. 2015), and diet plays a dominant role in shaping the ruminal microbial community and deciding the ruminal fermentation patterns. Concentrate-rich or forage-based diets are dominated by starch-degrading amylolytic bacteria or fibrolytic bacteria in rumen, which mainly degrade starch or fiber and produce a substantial amount of propionate or acetate, respectively. However, little information is available on the effect of physical corn processing method on the rumen microbial communities. Considering the increased performance and physical changes in heifers fed SFC, we hypothesized that differently processed corn modulated the ruminal bacterial community and the rumen metabolites, thereby improving animal performance. Therefore, the aim of this study was to investigate the ruminal bacterial community composition and fermentation parameters of heifers fed differently processed corn using 16S rRNA sequencing technologies, and further uncover the potential mechanisms by which the highly efficient SFC processing method aids ruminants.

## Materials and methods

### Animals, experimental design and sample collection

Twenty-six Holstein heifers (7 months old; body weight 291 ± 23 kg, mean ± SD) housed at a commercial dairy farm were selected for the experiment. Holstein heifers were blocked by weight and randomly assigned into two groups with thirteen heifers each. The experimental diets were steam-flaked corn (SFC) and finely ground corn (FGC) diet. Diets were formulated to meet the nutrient requirements for heifers (NRC 2001). The ingredients and nutritional composition of the diet are showed in Table 1. Heifers were fed twice a day at 0800 and 1600. Water and feed were provided for ad libitum consumption. The experiment was last for four weeks. All heifers were weighed 3 consecutive days at the beginning and the ending of the experiment periods. The rumen fluid was sampled at the last 2 consecutive days of the experimental periods. This study is part of our unpublished research, and the original experiment was a 2 × 2 factorial design with two factors of rumen escape starch levels (SFC and FGC diets) and rumen-protected leucine supplementation. The unpublished article mainly focuses on postruminal starch digestion, and rumen-protected leucine has no effect on rumen fermentation. Therefore, we organized the present study by focusing only on the main effect of rumen escape starch levels on rumen metabolites and microbes.

**Table 1:** Ingredients and chemical composition of the experimental diets (dry matter basis%)

Items	Treatments	
	SFG	FGC
Ingredient %		
Alfalfa hay	13.9	13.9
Oat	11.5	11.5
Corn silage	20.4	20.4
Steam flaked corn	39.8	
Finely ground corn		39.8
Soybean meal	11.2	11.2
Premix^a^	3.2	3.2
Total	100	100
Chemical composition		
Dry matter, %	55.5	55.8
CP, %	13.8	14.0
Starch, %	33.5	33.0
NDF, %	23.5	23.5
ADF, %	15.4	15.5

*SFC* steam flaked corn treatment, *FGC* finely ground corn treatment

^a^Each kilogram contained 1800 mg Fe, 350 mg Cu, 2160 mg Mn, 2660 mg Zn, 14 mg Se, 21 mg I, 18 mg Co, 180 000 IU vitamin A, 45 000 IU vitamin D and 2150 IU vitamin E

Rumen fluid was collected by oral stomach tubes at 2 h and 4 h post feeding on the sampling day, according to a reported procedure (Imhasly et al. 2014). After discarding the first 50 mL of fluid to minimize saliva contamination, approximately 100 mL of rumen sample was collected. Immediately after collection, 2 mL of the rumen sample was aliquoted and stored in liquid nitrogen for DNA extraction, and the rest of the rumen sample immediately measured pH and was filtered through four layers of sterile cheesecloth. The 10 mL filtered liquid sample was acidified with 2 mL of 25% (weight/volume) metaphosphoric acid and stored at − 20 °C until analysis of the ruminal fermentation characteristics. Before sample analysis, the samples taken from the two time points were mixed together.

### Ruminal metabolite analysis

Ruminal VFA (including acetate, propionate, butyrate, valerate, isobutyrate and isovalerate) concentrations of each sample were analyzed by gas chromatography (Agilent Technologies 7820A GC system, Santa Clara, USA) using a 30 m × 0.25 mm × 0.33 μm fused silica column (AE-FFAP; ATECH Technologies Co., Ltd, Lanzhou, China) after removing the solid particles and proteins in the sample according to previously described methods (Li et al. 2014). Ruminal ammonia nitrogen (NH_3_-N) was measured as previously described (Chaney and Marbach 1962).

### Feed chemical analyses

Feed samples were dried for 24 h at 105 °C for dry matter (DM) analysis to adjust dietary ingredient inclusion rates. Duplicate composite samples were dried in a hot-air oven at 55 °C for 48 h, ground in a Wiley mill with a 2-mm mesh screen (Thomas-Wiley Laboratory Mill) followed by a 1-mm mesh screen. All the samples were analyzed for DM (105 °C for 8 h), CP (method #988.05) and crude ash (#942.05) according to the AOAC Methods (AOAC International 2002). Diet NDF and ADF were analyzed with sodium sulfite and heat-stable α-amylase (Ankom A200I Fiber Analyzer, NKOM Technology, Macedon, NY, USA) according to Van Soest et al. (1991). Starch content was determined by an enzymatic method (α-amylase and amyloglucosidase) with a commercial starch analysis kit (Megazyme International Ireland Ltd., Bray, Ireland).

### DNA extraction, PCR amplification and Illumina MiSeq sequencing

The full-length 16S rRNA genes of all rumen fluid samples in this study were assessed using high-throughput sequencing on the PacBio ^®^ RS II platform (Majorbio Bio-Pharm Technology Co., Ltd., Shanghai, China). Genomic DNA was extracted from ruminal samples using the E.Z.N.A.^®^ soil DNA Kit (Omega Bio-tek, Norcross, GA, U.S.) according to the manufacturer’s protocols. The final DNA concentration and purity were determined by a NanoDrop 2000 UV–Vis spectrophotometer (Thermo Scientific, Wilmington, USA), and DNA quality was checked by 1% agarose gel electrophoresis. The V3-V4 hypervariable regions of the bacterial 16S rRNA gene were amplified with primers 338F (5′-ACTCCTACGGGAGGCAGCAG-3′) and 806R (5′-GGACTACHVGGGTWTCTAAT-3′) and a thermocycler PCR system (GeneAmp 9700, Applied Biosystems, Foster City, CA, USA). The PCR reactions were conducted using the following program: 3 min of denaturation at 95 °C, 27 cycles of 30 s at 95 °C, 30 s for annealing at 55 °C, and 45 s for elongation at 72 °C, and a final extension at 72 °C for 10 min. PCR reactions were performed in triplicate 20 μL mixture containing 4 μL of 5 × FastPfu Buffer, 2 μL of 2.5 mM dNTPs, 0.8 μL of each primer (5 μM), 0.4 μL of FastPfu Polymerase and 10 ng of template DNA. The resulted PCR products were extracted from a 2% agarose gel and further purified using the AxyPrep DNA Gel Extraction Kit (Axygen Biosciences, Union City, CA, USA) and quantified using QuantiFluor™-ST (Promega, Madison, WA, USA) according to the manufacturer’s protocol. Purified amplicons were pooled in equimolar and paired-end sequenced (2 × 300) on an Illumina MiSeq platform (Illumina, San Diego, USA).

### Sequencing data processing

Raw fastq files were demultiplexed, quality-filtered by Trimmomatic (Bolger et al. 2014) and merged by FLASH (Magoč and Salzberg 2011) with the following criteria: (i) The reads were truncated at any site receiving an average quality score < 20 over a 50 bp sliding window. (ii) Reads exactly matching primers, allowing 2 nucleotide mismatching, and reads containing ambiguous bases were removed. (iii) Sequences whose overlap was longer than 10 bp were merged according to their overlap sequence. Operational taxonomic units (OTUs) were clustered with 97% similarity cutoff using UPARSE(version 7.1 http://drive5.com/uparse/), and chimeric sequences were identified and removed using UCHIME. The taxonomy of each 16S rRNA gene sequence was analyzed by the RDP Classifier algorithm (http://rdp.cme.msu.edu/) against the Silva (SSU123) 16S rRNA database using a confidence threshold of 70%. The identified sequences were deposited in the NCBI sequence archive (SRA) under accession no. PRJNA552771.

### Predicted molecular functions based on 16S rRNA data using PICRUSt

To explore the functional profiles of our bacterial community data set, we used PICURSt to predict gene family abundances based on 16S rRNA data. PICRUSt uses an extended ancestral-state reconstruction algorithm to predict which gene families are present and then combines gene families to estimate the composite metagenome (Langille et al. 2013). The available annotated genes were imputed into Kyoto Encyclopaedia of Genes and Genomes (KEGG) (http://www.genome.ad.jp/kegg/) to predict metagenomics potential. The predicted genes and their functions were aligned to KEGG database. The principal component analysis (PCA) plotting and statistical hypothesis tests for pairs of samples were then performed using STAMP software (Parks et al. 2014).

### Statistical analysis

The microbiota OTU data, phylum and genus relative abundances, rumen VFA (acetate, propionate, butyrate, isobutyrate, valerate, isovalerate, acetate to propionate ratio and total VFAs) concentrations, NH_3_-N concentration, rumen pH and average daily gain (ADG) were analyzed using the one-way ANOVA of SPSS software (Version 18.0; IBM SPSS, Armonk, NY). In addition, one-way ANOVA of the microbiota data was based on the normal distribution test. Treatment differences with *P* < 0.05 were considered statistically significant, and 0.05 < *P* < 0.10 was designed as a tendency. Principal coordinate analysis (PCoA) and analysis of similarities (ANOSIM) were analyzed with the FactoMine and vegan package in R software (http://www.R-project.org/). Correlations between bacterial taxa and rumen fermentation characteristic variables were calculated by non-parametric Spearman’s rank correlation analysis using R software.

## Results

### Changes in rumen microbial metabolites and the growth performance of heifers

**Fig. 1:**
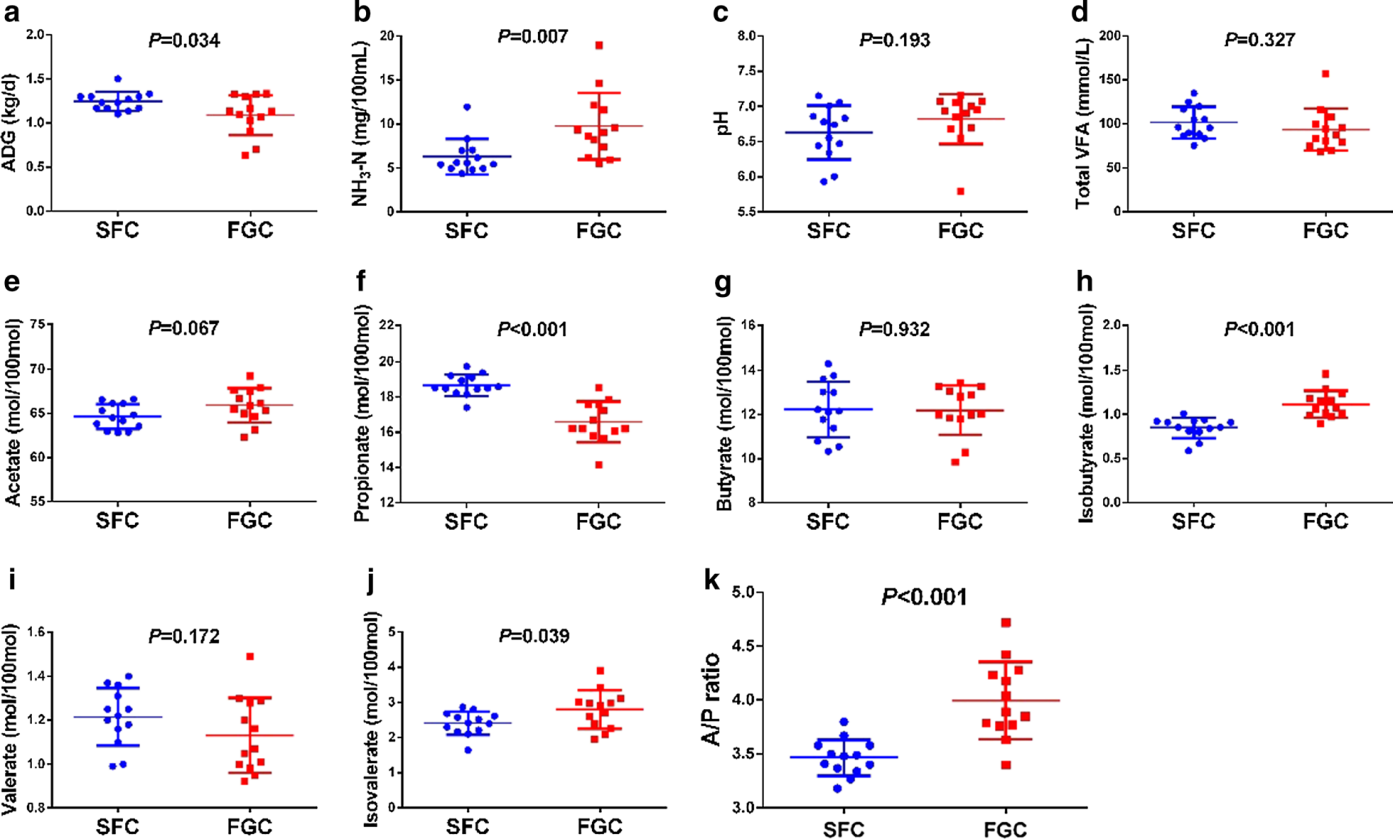
Effects of different corn processed methods (steam flaked corn (SFC) and finely ground corn (FGC)) on rumen fermentation characteristics and growth performance of dairy heifers. **a** average daily gain; **b** NH_3_-N in rumen fluid; **c** pH in rumen fluid; **d** total VFA; **e** acetate; **f** propionate; **g** butyrate; **h** isobutyrate; **i** valerate; **j** isovalerate; **k** acetate-to-propionate ratio

In the present study, corn processing changed rumen microbial metabolites and growth performance in dairy heifers. First, the ADG was significantly higher in the SFC group than the FGC group (*P *= 0.034; Fig. 1a), as expected. The concentration of total rumen VFA was similar among treatments (*P *= 0.327; Fig. 1d). However, the propionate proportion was markedly elevated when heifers were fed the SFC diet (*P *< 0.001; Fig. 1d). In contrast, the proportion of isobutyrate (*P *< 0.001; Fig. 1d), isovalerate (*P *= 0.039; Fig. 1d) and the acetate-to-propionate ratio (*P *< 0.001) were significantly decreased when heifers were fed the SFC diet. The acetate proportion tended to be lower in the heifers fed the SFC diet than in the heifers fed the FGC diet (*P *= 0.067; Fig. 1d). The ruminal pH was not affected by the different corn processing methods (*P *= 0.193; Fig. 1d). The SFC diet significantly reduced the NH_3_-N production (*P *= 0.007; Fig. 1d).

### Assessment of the population diversity of the ruminal bacterial

A total of 1,507,527 high-quality DNA sequences were obtained from 26 rumen fluid samples. The average length of the sequence reads was 434 bp. High-quality reads were clustered using > 97% sequence identity into 1260 microbial OTUs. Alpha diversity indices indicted that the SFC diet significantly decreased the Shannon index (*P *= 0.010) and increased the Simpson index (*P *= 0.018) (Table 2).

**Table 2:** Alpha diversity indices for species richness, abundance, and population diversity in the rumen bacterial communities in heifers fed different corn processed corn diet

Items	Treatments		SEM	*P* value
	SFC	FGC		
Ace	7.66	10.16	0.826	0.134
Chao	9.69	10.39	0.269	0.204
Shannon	0.92	1.06	0.029	0.010
Simpson	0.50	0.43	0.016	0.018
Sob	9.62	10.31	0.251	0.173

*SFC* steam flaked corn treatment, *FGC* finely ground corn treatment

**Fig. 2:**
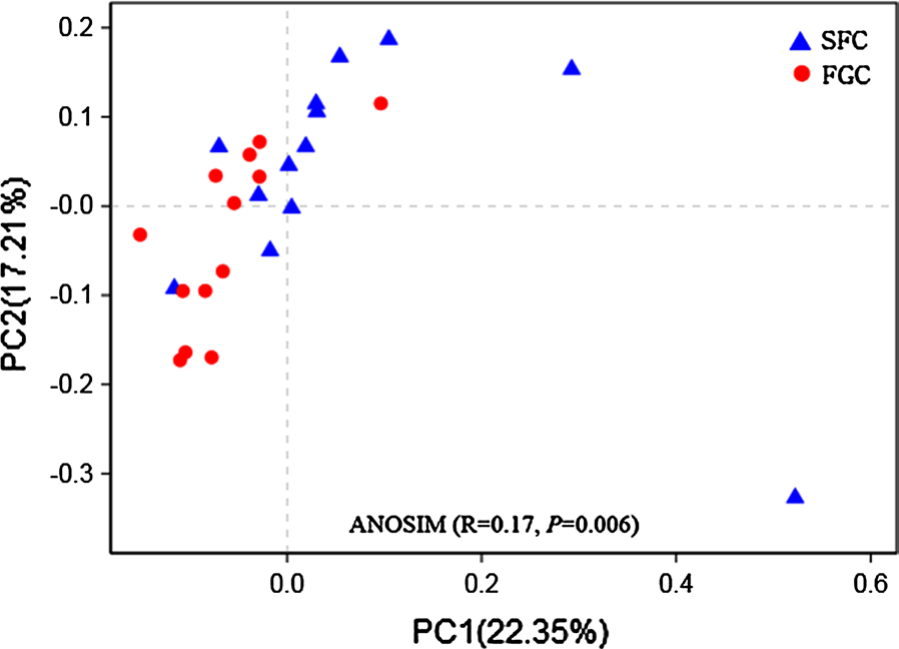
Principal coordinate analysis (PCoA) of rumen bacterial beta diversity for heifers fed steam flaked corn (SFC) or finely ground corn (FGC)

For further analysis of the difference in bacterial community diversity, PCoA based on unweighted UniFrac distance metrics and ANOSIM were conducted. The PCoA plots revealed a clear clustering between samples from the SFC and FGC groups (Fig. 2), and the result of ANOSIM demonstrated that this difference reached a statistically significant level (R = 0.17, *P *= 0.006).

### Ruminal microbiota composition

At the phylum level, 14 phyla were identified in the rumen samples. Among them, *Firmicutes* and *Bacteroidetes* were the dominant phyla and accounted for 60.43% and 27.27% of the total sequences, respectively, followed by *Actinobacteria* (5.67%), *Spirochaetae* (4.34%), *Tenericutes* (1.25%) and *Proteobacteria* (0.62%) (Additional file 1: Table S1). At the genus level, 214 classifiable genera were detected in all samples. The dominant genera were *Ruminococcaceae_UCG*-*005* (19.99%) and *Bacteroides* (7.14%), followed by *norank_f__Bacteroidales_S24*-*7_group* (6.20%), *Rikenellaceae_RC9_gut_group* (5.46%), *Bifidobacterium* (5.12%), *unclassified_f__Lachnospiraceae* (4.89%), *Treponema_2* (4.34%) and *Prevotellaceae_UCG*-*003* (3.04%) (Supplementary Table S2).

### The ruminal microbiota differed between heifers fed the SFC and FGC diets

**Fig. 3:**
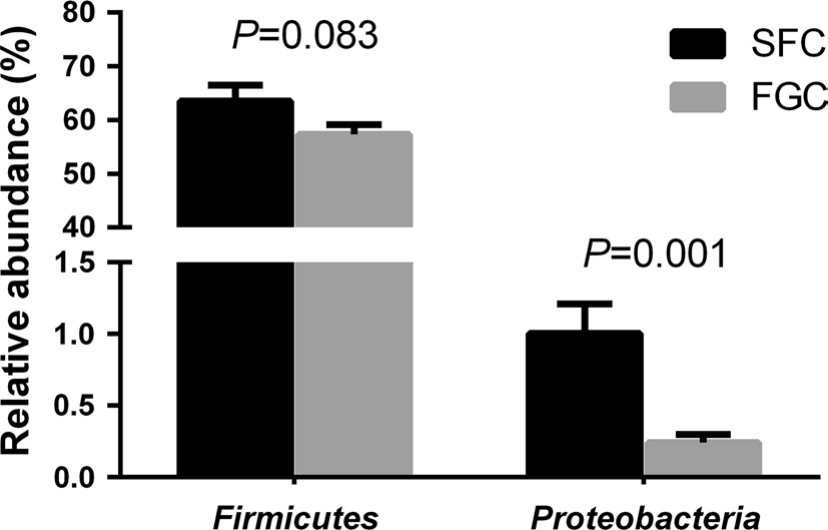
Comparison of the distribution of rumen bacteria with significant effects at the phylum level for heifers fed steam flaked corn (SFC) or finely ground corn (FGC) diets based on 16S rRNA sequences compared to the SILVA version 128 database. Error bars indicate the standard error of the mean

The relative abundances of ruminal bacteria at the phylum level revealed the significant effects of corn processing methods on *Firmicutes* and *Proteobacteria* (Fig. 3). The SFC diet significantly increased the relative abundance the phylum *Proteobacteria* (*P *= 0.001) and tended to increase *Firmicutes* (*P* = 0.083). However, no significant differences were observed in other phyla, including the second most abundant phyla *Bacteroidetes* (*P* = 0.907) between the SFC and FGC diets.

Separation and analysis of the sequencing data were also performed at the genus level, with a total of 10 genera affected by the corn processing methods (Table 3). Of these, 7 genera belonging to *Firmicutes* were found, including *Roseburia* (*P* = 0.037), *Blautia* (*P* < 0.001), *Marvinbryantia* (*P* = 0.011), *[Ruminococcus]_gauvreauii_group* (*P* = 0.027) and *unclassified_o__Clostridiales* (*P* = 0.066), which were significantly higher or tended to be higher in heifers fed the SFC diet than FGC diet. In contrast, *Ruminococcaceae_UCG*-*014* (*P* = 0.036) and *Ruminococcaceae_UCG*-*013* (*P* = 0.002) were decreased in heifers with the SFC diet. The SFC diet significantly increased the relative abundance of *Succinivibrio* (phylum *Proteobacteria*) and tended to increase *norank_f__Bacteroidales_S24*-*7_group* (phylum *Bacteroidetes*), but decreased the relative abundance of *Alistipes* (*P* = 0.003, 0.091 and 0.022, respectively).

**Table 3:** Percent relative abundance of genera with a significant effect of diet with steam flaked corn (SFC) or finely ground corn (FGC)

Items^a^	Treatments		SEM	*P* value
	SFC	FGC		
*Bacteroidales_S24*-*7_group*	7.24	5.15	0.616	0.091
*Roseburia*	2.85	1.55	0.314	0.035
*Blautia*	3.34	0.75	0.374	< 0.001
*Ruminococcaceae_UCG*-*014*	0.95	1.66	0.169	0.033
*Alistipes*	0.62	1.54	0.207	0.022
*Marvinbryantia*	1.11	0.69	0.087	0.013
*Ruminococcaceae_UCG*-*013*	0.31	0.96	0.110	0.002
*[Ruminococcus]_gauvreauii_group*	0.77	0.36	0.094	0.027
*Unclassified_o__Clostridiales*	0.53	0.30	0.061	0.058
*Succinivibrio*	0.75	0.04	0.125	0.002

^a^Relative abundance ≥ 0.1%

### Association of ruminal bacteria and metabolites

**Fig. 4:**
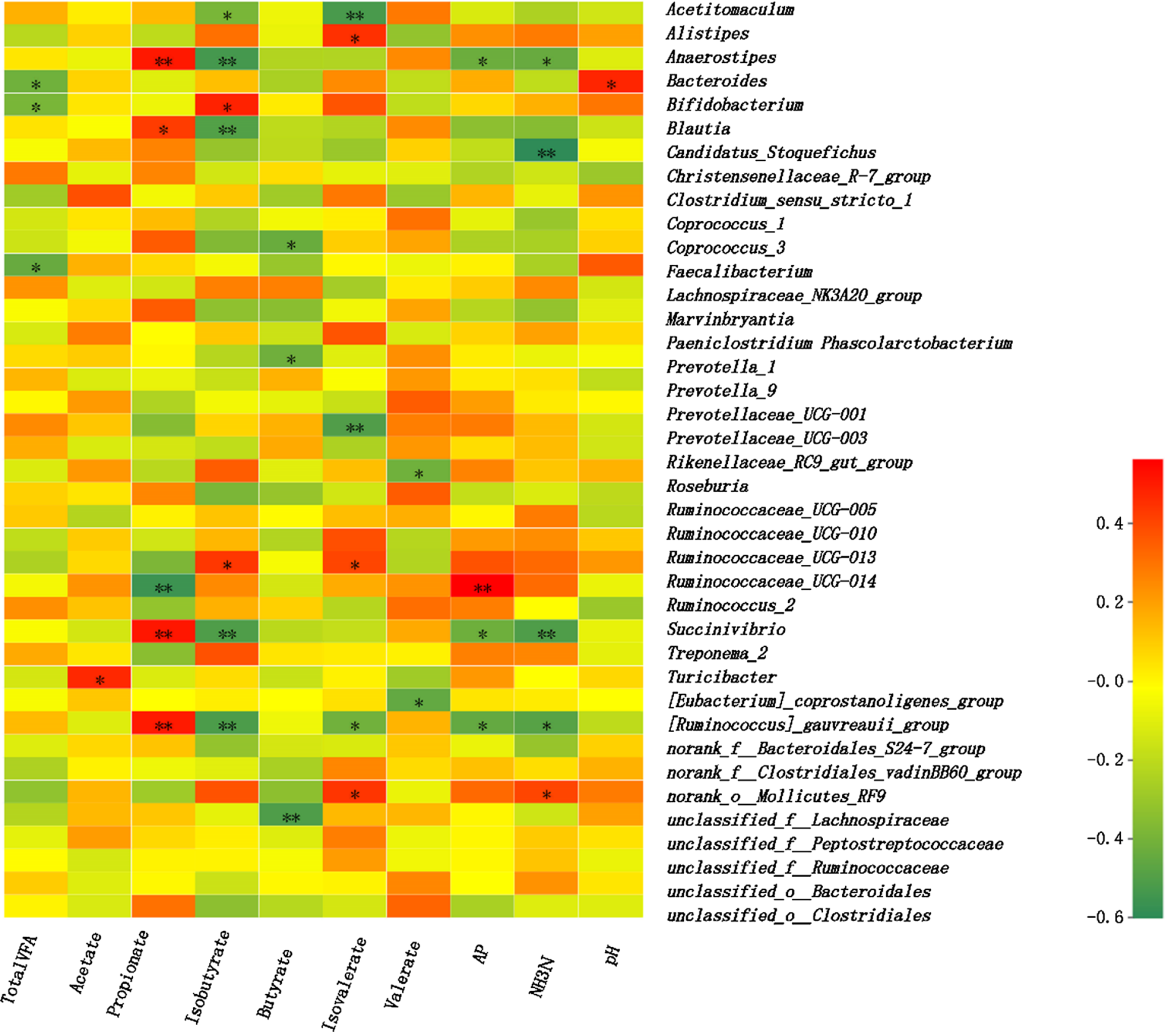
Correlation between the relative abundances of bacterial genus level and the fermentation characteristics (top 40 genus). Total VFA, total volatile fatty acid; AP, acetate-to-propionate ration; AN, NH_3_-N. * means 0.01 < *P* ≤ 0.05, **means 0.001 < *P* ≤ 0.01

To investigate the correlation between metabolites and the changes in the rumen microbiota in different groups, we conducted an association analysis (Spearman’s rank correlation coefficients) of the phenotypic module to detect significantly different rumen microbiota. Furthermore, total VFA, acetate, propionate, isobutyrate, butyrate, isovalerate, valerate, acetate-to-propionate ratios, NH_3_-N, and pH were correlated with the rumen microbiota. Unless otherwise indicated, significant correlations are considered a *P* value of ≤ 0.05, whereas highly significant results are considered a *P* value of ≤ 0.001. As shown in Fig. 4, the results are summarized in a heatmap. The correlation analysis showed that the relative abundance of *Succinivibrio* was positively correlated with propionate, but negatively correlated with NH_3_-N. *Bacteroides*, *Bifidobacterium*, and *Faecalibactrium* were negatively correlated with total VFA. *Turicibacter* was positively correlated with acetate. *Blautia* and *[Ruminococcus]_gauvreauii_group* were positively correlated with propionate, but *Ruminococcaceae_UCG_014* was negatively correlated with propionate. In addition, *Coprococcus_3*, *Phascolarctobacterium* and *unclassified_f_Lachnospiraceae* were negatively correlated with butyrate. *Rikenellaceae_RC9_gut_group* and *[Eubacterium]_coprostanoligenes_group* were negatively correlated with valerate. *[Ruminococcus]_gauvreauii_group* was negatively correlated with NH_3_-N.

### Predicted functional profiles of the rumen bacterial community

**Fig. 5:**
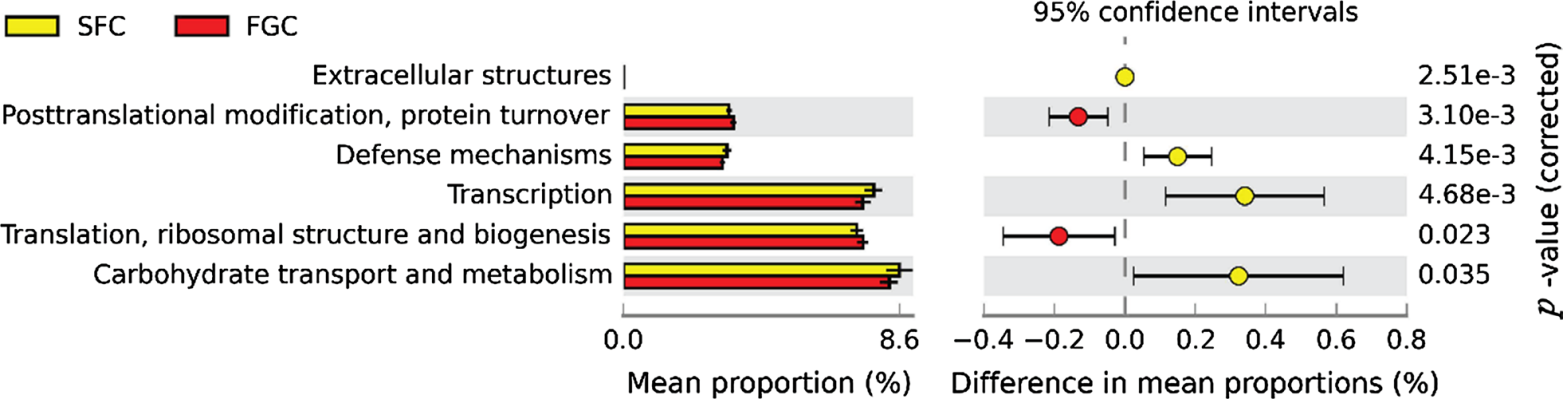
Comparison of the distribution of the abundance of Kyoto Encyclopedia of Genes and Genomes (KEGG) pathways of rumen bacterial community for heifers fed steam flaked corn (SFC) or finely ground corn (FGC) diets. Only the significantly affected KEGG pathways by dietary treatments are shown

The functional profile of the rumen bacterial community was predicted using PICRUSt using level 3 of KEGG orthologs. As shown in Fig. 5, compared with the FGC diet, the SFC diet increased the abundance of carbohydrate transport and metabolism (*P* = 0.034), transcription (*P* = 0.005), extracellular structures (*P* < 0.001) and defense mechanisms (*P* = 0.003), whereas it decreased the abundance of translation, ribosomal structure and biogenesis (*P* = 0.022) and posttranslational modification, protein turnover, and chaperones (*P* = 0.003).

## Discussion

Dietary composition plays a predominant role in determining both the community structure and the metabolic function of the rumen microbiota (Petri et al. 2013). However, the influence of different physical corn processing methods on the rumen bacterial community composition and their relationship with ruminal metabolites remain largely unknown. In particularly, ruminants usually perform better when fed SFC diets than FGC diets. The steam flaking processing method results in a more complete gelatinization than the grinding method and increases starch availability (Svihus et al. 2005), further significantly increasing ruminally degradable starch (Davies et al. 2013; Shen et al. 2015; Luo et al. 2017). Therefore, we hypothesized that the steam flaking processing method may modulate microbial fermentation due to the changes in the patterns of substrate availability for rumen microbiota.

When fermented, fiber, starch and sugar could yield VFA (Owens and Basalan 2016). Analyses of the energetic efficiency of metabolized VFA from glucose have shown that propionate is the most efficient one, followed by butyrate and acetate (Ryle and Ørskov 1990). In the present study, a similar total VFA concentration in heifers fed SFC and FGC was observed. Consistent with our study, the total rumen VFA production was similar to animals fed FGC or SFC (Corona et al. 2005; Shen et al. 2015). The SFC diet provided higher propionate concentrations in the rumen than the FGC diet, which led to a lower acetate-to-propionate ratio in heifers fed SFC diet. A potential explanation for this result is that the starch in corn experiences different degrees of exposure to enzymatic attack in the rumen (Beauchemin et al. 1994; Huntington 1997). In addition, a previous study reported that the propionate concentration and the propionate-to-acetate ratio in efficient cows were significantly higher than those in inefficient groups (Shabat et al. 2016), which indicated that steam-flaking corn is a more efficient processing method than grinding corn for cows. Moreover, the increased rumen propionate concentration could decrease the hydrogen availability for methanogenesis and increase the precursors available for gluconeogenesis in animals, ultimately improving feed efficiency (Li and Guan 2017; Li et al. 2018). This may explain why the SFC diet performed better than the FGC diet, as supported by the SFC group having a greater ADG than the FGC group in our study. The ruminal NH_3_-N concentration was lower in heifers fed the SFC diet than those fed the FGC diet, which was reported in a previous study (Zhong et al. 2008; Davies et al. 2013). In addition, the reduced ruminal NH_3_-N concentration in the SFC group could be related to the improved efficiency of microbial crude protein synthesis (Shen et al. 2015), because the dietary energy and nitrogen were synchronously supplied for rumen metabolism.

Rumen metabolite changes are the outcome of microbiome structures, because many studies have shown that the alterations in the microbial community affects microbial metabolites. Therefore, the microbial structure was further studied. The SFC diet was associated with a decrease in community diversity, with a reduced Shannon index and increased Simpson index. It’s consistent with previous studies that low richness and diversity tends to be associated with host feed efficiency (Shabat et al. 2016), and also the low richness and diversity has been reported to be related to increased energy harvest from feed in obese humans (Turnbaugh et al. 2009; Le Chatelier et al. 2013).

As indicated by the separation of sample points from the two groups in the PCoA plots and ANOSIM, the composition of the rumen bacterial community was also significantly diverse. Generally, the changes were mainly reflected by an increase in the relative abundance of the phyla *Firmicutes* and *Proteobacteria*, genera *Bacteroidales_S24*-*7_group*, *Roseburia*, *Blautia*, *Marvinbryantia*, *[Ruminococcus]_gauvreauii_group*, *Unclassified_o__Clostridiales* and *Succinivibrio* and a decrease in genera *Ruminococcaceae_UCG*-*014*, *Alistipes* and *Ruminococcaceae_UCG*-*013*. The numbers of the phylum *Firmicutes* are involved in the degradation of starch (Kaoutari et al. 2013). The relative abundance of *Proteobacteria* was increased linearly in dairy heifers fed increasing dietary concentration levels (Zhang et al. 2018a), or it was found at relatively higher levels in cattle offered high concentrates diets (Auffret et al. 2017). The alteration in the genus *Succinivibrio* (phylum *Proteobacteria*) was also observed in this study, and *Succinivibrio* appears to be a major fermenter of dextrins (Bryant 2015). The abundance of *Succinivibrio* is usually relatively higher when cattle or sheep are fed high-grain diets containing large amounts of starch or rapidly fermentable carbohydrates (Bryant and Small 1956; Wozny et al. 1977; Kim et al. 2014; Henderson et al. 2015; Plaizier et al. 2017; Zhang et al. 2018b). Interestingly, an increased abundance of *Succinivibrio* species in the rumen was linked to the reduced methane emissions in cattle (Holman and Gzyl 2019). It has been suggested that *Succinivibrio* could utilize hydrogen to synthesize succinate, therefore lowering the amount of hydrogen available to methanogens for methane production, and succinate can be further decarboxylated to form propionate (Hespell 1992; Pope et al. 2011). Consistent with the ruminal microbial metabolites, the SFC diet significantly increased the propionate proportion, and the correlation analysis showed that the abundance of *Succinivibrio* was significantly positively correlated with the propionate concentration. For the genus *Roseburia*, which could hydrolyze and ferment starch (Stanton et al. 2015), the abundance was increased linearly with increasing dietary concentration levels (Zhang et al. 2018a). *Blautia* utilizes carbohydrates as fermentable substrates (Park et al. 2013), and its abundance was positively correlated with the propionate concentration in our study. Thus, we suggest that *Blautia* might ferment starch and produce propionate as an end product. Additionally, *Blautia* was noted as the only changed genus related to steer’s feed efficiency, and steers with greater ADG had greatest abundance of it (Myer et al. 2015). The increased *Blautia* abundance may be also related to the increased milk efficiency in dairy cows fed an SFC diet (Guyton et al. 2003; Cooke et al. 2008). *Blautia* can mediate beneficial anti-inflammatory effects on acute graft-versus-host diseases (Jenq et al. 2015), which is possible to explain why *Blautia* aided host. When examining the association between bacterial community and the utilization efficiency of nitrogen in goats, the abundance of *Bacteroidales_S24*-*7_group* was observed higher in the high-nitrogen-utilization phenotype group than in the low-nitrogen-utilization phenotype group (Wang et al. 2019). In our study, the NH_3_-N concentration was much lower in the SFC group, which indicated that the *Bacteroidales_S24*-*7_group* genus efficiently converted non-protein nitrogen to microbial proteins and improved microbial protein synthesis. Moreover, the PICRUSt predictions results also showed that the genes responsible for carbohydrate transport and metabolism, defense mechanisms and transcription were upregulated in the SFC diet. This may explain the fact that the SFC diet increased the amount of starch digested in the rumen and thereby improved the ruminal nitrogen utilization efficiency.

Corresponding to the increase in amylolytic bacteria, the relative abundance of cellulolytic bacteria was decreased with the SFC diet. As expected, the abundances of *Ruminococcaceae_UCG*-*014* and *Ruminococcaceae_UCG*-*013*, which are genera defined as cellulose- and hemicellulose-degrading bacteria within the rumen (Flint et al. 2008; Singh et al. 2014), were significantly decreased with the SFC diet. *Ruminococcus* is considered to be the most important fiber-degrading bacterium in the intestine of herbivores, and produces large amounts of cellulolytic enzymes, including exoglucanases, endoglucanase, glucosidases and hemicellulases (Singh et al. 2014). In contrast to *Blautia*, the correlation analysis of the genera and rumen metabolites showed that *Ruminococcaceae_UCG*-*014* was negatively correlated with the propionate concentration, which is the main end product of starch fermented by amylolytic bacteria. In addition, the genus *Alistipes* might also be specifically involved in fiber degradation (Peng et al. 2015). In a previous study, high abundances of *Alistipes* and *Ruminococcus* in buffalo and cattle were associated with fiber degradation (Zhang et al. 2017). The additional functions of these unclassified bacteria are not clear, and hence, more research is needed to determine their roles in the process of starch and fiber degradation. Such studies will help us to fully understand the mechanisms underlying the different animal performances between those fed SFC and FGC diets. However, with the current limited availability of knowledge, we can infer that the increased rumen propionate concentration produced by amylolytic bacteria contributes to the increased efficiency of dairy heifers with high ADG when fed the SFG diet.

In summary, our results suggested that steam flaked corn promoted the increased abundance of amylolytic bacteria, especially the genus *Succinivibrio*, thereby increasing propionate production. Propionate is the highest energetic efficiency metabolite for ruminant hosts, and thus, dairy heifers with increased propionate concentration exhibited relatively higher average daily gain. This study provides comparative evidence for the mechanism underlying the enhanced performance of ruminants fed steam flaked corn.

## Supplementary information

**Additional file 1: Table S1.** Summary of the relative abundance of phyla; **Table S2.** Summary of the relative abundance of genera.

## Data Availability

We declared that materials described in the manuscript, including all relevant raw data, will be freely available to any scientist wishing to use them for noncommercial purpose, without breaching participant confidentiality.

## Abbreviations

SFC: steam flaked corn
FGC: finely ground corn
VFA: volatile fatty acid
NH_3_-N: ammonia nitrogen
ADG: average daily gain
OUT: operational taxonomic unit
KEGG: Kyoto Encyclopedia of Genes and Genomes
